# Mimicry Enhances Observational Learning in 16-Month-Old Infants

**DOI:** 10.1371/journal.pone.0113695

**Published:** 2014-12-10

**Authors:** Eszter Somogyi, Rana Esseily

**Affiliations:** 1 Laboratoire Psychologie de la Perception, Université Paris V, CNRS UMR 8158, Centre Biomédical des Saints-Pères, 75006, Paris, France; 2 Laboratoire Éthologie, Cognition, Développement, Université Paris Ouest Nanterre la Défense, EA 3456, 92000, Nanterre, France; Utrecht University, Netherlands

## Abstract

We examined the effect of mimicry on how 16-month-old infants learn by observation a novel tool use action, which consisted of using a rake to retrieve a toy. Across four conditions, we manipulated whether during an initial play phase, an adult mimicked the infant's play or not (testing the effect of mimicry), the infant played with the adult or played alone (controlling the effect of interacting with a contingent partner) and whether the infant saw a demonstration of the tool's use or not (evaluating baseline performance). We found that infants who had been mimicked learned best from a demonstration of the rake's use and performed better than infants who only played with the experimenter without mimicry or played by themselves before the demonstration. As expected, infants did not learn from a demonstration of the rake's use when they played by themselves and thus had no previous interaction with an experimenter. The mechanisms driving this powerful learning effect of mimicry are discussed.

## Introduction

The goal of this study was to investigate whether mimicry has an impact on infants' social learning, in particular whether it facilitates observational learning of a new tool use action. Mimicry is a special type of imitation, also referred to as synchronous imitation in infants and defined as the systematic overt imitation of each other's behaviour, generally in the context of a playful interaction between peers [1]. In adults, mimicry becomes subtle and refers rather to the automatic and non-conscious imitation of others' facial expressions, postures, gestures, mannerisms or verbal behaviours and has been coined the ‘chameleon effect’ [2]. Mimicry has an important role in our interactions, enabling pleasant and smooth social exchange. With mimicry, we facilitate interpersonal affiliation [2]–[5], perception of empathy [6] and influence others to become more prosocial (see [7], [8] for adults and [9] for an infant study). In infants, mimicry has also been shown to encourage initiation of subsequent joint interactions [10].

Parents imitate infants spontaneously and naturally in everyday situations [11], [12]. Infants in turn detect and appreciate mirroring behaviours already at 2 months, responding with more attention, smiling, and positive vocalizations [1]. By 9 months, infants reliably distinguish and prefer mimicry over temporally contingent behavior [13], [14] and by 14 months of age they engage in systematic testing behaviours, by, for instance, modulating their own actions on a toy while looking at the adult to check whether he or she is intentionally mimicking [13], [15]. At 16 months, toddlers begin to mimic- or synchronously imitate each other during natural play and mimicry becomes a widely applied behavioural strategy with peers. Throughout the following months, mimicry emerges as a pre-linguistic form of communication [1], with a peak around 30 months of age [16], [17].

It is therefore clear that infants are interested in an adult or peer's matching behaviour and that they use mimicry to communicate. But does mimicry also have consequences regarding infants' general social behaviour? Two studies investigated this question by experimentally manipulating mimicry during play in 18-month-olds. Fawcett and Liszkowski [10] examined the effect of mimicry on the initiation of subsequent joint interactions. In an initial play phase, infant and experimenter had identical sets of toys to play with. In the mimicry condition the experimenter mimicked all the infant's actions on the toys, whereas in the no mimicry condition she played with her own toys during the same amount of time and did not mimic the infants' actions. Following this first 4-minute play phase, infants initiated play more frequently with the mimicking adult, but this increase in inviting behavior did not generalize to other individuals. Thus mimicry served as a non-verbal form of committing to joint interaction. In the second study Carpenter and colleagues [9] investigated whether being mimicked increases prosocial behavior in 18-month-old infants. In their procedure, infants were mimicked while looking at a series of pictures with the experimenter and while they were freely exploring the room afterwards. In the mimicry condition, the experimenter copied all the infants' actions immediately, whereas in the no mimicry condition, the experimenter contingently performed different, but still natural and friendly actions. In two subsequent helping tests infants were given the opportunity to help either the same or a different adult to pick up some sticks that fell to the floor or to open a cabinet. Infants who had previously been mimicked were significantly more likely to help the adult than infants to whom the adult responded in a temporally contingent way, but without mimicry. Being mimicked also increased infants' willingness to help a different adult who had not been involved in the mimicry situation at all. Thus, just like in adults [7], mimicry increased general prosocial behaviour.

As noted above, mimicry in infant studies [9], [10], [17] is much more overt and immediate than the unconscious, subtle, and slightly delayed bodily mimicry studied in adults. Indeed, in adults and older children, obvious mimicry can be perceived negatively, whereas infants appreciate it. This can be explained by the above-mentioned transitory communicative function of mimicry in preverbal infants, who use imitation as an explicit form of intentional communication [1], [18]–[20]. In fact, it has been shown that both forms of mimicry, subtle and explicit, serve similar functions in adults and children, such as promoting affiliation [5], [21] or countering exclusion from group [22].

If mimicry is such a powerful social tool, does it also enable learning in preverbal children?

Only one study investigated the effects of mimicry on learning in infants. In their study described above, Fawcett and Liszkowski [10] also compared how infants reproduced a series of action steps in the mimicry and the no mimicry conditions. They only found a marginally significant effect; infants reproduced about equal numbers of actions steps in the two conditions. This result, however, might be due to the simplicity of the task, which involved mostly affordant, easy-to-perform action steps that were probably not new to 18-month-olds (choosing the same tool as the experimenter, tapping the tool on the base of the toy, knocking posts over with the tool, and replacing the posts with the hand). As there was no baseline group to test infants' spontaneous actions on the tools and toys involved in this trial, we cannot exclude the possibility that the trial did not involve social learning. In other words infants could have produced the same actions without any demonstration, thus without learning these from the experimenter. The aim of the present study was therefore to investigate the effect of being mimicked on social learning in a more controlled setting where infants' learning can be measured more reliably.

A further concern with studies on the effect of infant mimicry is the effect of play. Mimicry can be considered as an imitative game and its effects might therefore be driven by the playfulness of the situation. Indeed, Nielsen et al. [23] have shown that engagement with an adult who responds in a socially contingent way increases 24-month-olds' imitative behaviour. Everyday social contingency involves both temporal and spatial modalities, although in a less exaggerated manner than in the case of mimicry. Two of the studies cited above have controlled temporal contingency with conditions where the adult responded to the infants' actions immediately, but by producing a different action [9], [14]. These have shown that temporal contingency in itself does not explain the preference of a mimicking adult in 14-month-olds [14] or the social effects of mimicry in 18-month-old infants [9]. None of the studies so far, however, have controlled the effect of natural social contingency or compared its effects to those of mimicry. In order to do so, we included a control condition where the experimenter engaged into socially contingent interaction with the infants by playing with them.

We chose to study 16-month-old infants, since this is the age when toddlers start to imitate each other during natural play [16], [17] and by this age they reliably recognise and test mimicry [13], [15].

We tested the effect of mimicry on infants' observational learning in a tool use task where infants are required to use a rake in order to retrieve an out-of-reach object (Rake Task). Previous studies indicate that infants at this age do not succeed in this task even following a demonstration of the rake's use [24], [25]. Therefore any improvement we measured in our study could reliably be attributed to the effect of the absence or presence of mimicry across our experimental conditions. To confirm this assumption, we included a baseline control group where infants did not see a demonstration.

In order to enable mimicry and the perception of being imitated, we used an experimental setting comprising two sets of identical objects, which has been shown to facilitate mimicry and recognition of being imitated [1], [17].

Taken together, across our four experimental conditions, we manipulated the (a) presence or absence of mimicry before demonstration, the (b) presence or absence of play with a partner before demonstration and the (c) presence or absence of demonstration in the following way:

- (1) Mimicry + Demo Condition: infants played with an experimenter and were mimicked before observing a demonstration of the rake's use
- (2) Non-Mimicry + Demo Condition: infants played with an experimenter, but were not mimicked before demonstration
- (3) Play Alone + Demo Condition: infants played by themselves before demonstration
- (4) Play Alone + No Demo Condition: in this baseline condition, infants played by themselves and were tested directly on the Rake Task, without a demonstration of the rake's use.

Our first hypothesis was that mimicry would have a specific facilitating effect on infants' observational learning. We expected that infants who were mimicked beforehand (Mimicry + Demo Condition) would benefit best from a demonstration of the rake's use across our conditions and subsequently perform better on the Rake Task than infants who played with the experimenter for the same amount of time (Non-Mimicry + Demo Condition) or played by themselves (Play Alone + Demo Condition).

Our second hypothesis was that play with the experimenter would also facilitate infants' observational learning, but to a lesser extent. We therefore expected that infants who played with the experimenter (Non-Mimicry + Demo Condition) would perform better on the Rake Task than infants who played by themselves (Play Alone + Demo Condition), but not as well as infants who were mimicked beforehand (Mimicry + Demo Condition).

Thirdly, we expected to replicate previous results showing that 16-month-old infants do not succeed in the Rake Task either spontaneously or after a demonstration done without previous interaction with the experimenter [24], [25]. We anticipated that infants who saw a demonstration of the rake's use (Play Alone + Demo Conditions) would perform similarly on the Rake Task to infants in our baseline group who did not see a demonstration (Play Alone + No Demo Condition).

## Method

### 1. Participants

Forty-eight infants (mean age  = 492 days; range  = 16 months +/- 10 days; 22 females) participated in the study. Ten additional infants were excluded for the following reasons: lack of interest in the objects proposed either during initial play phase (n = 5 of which two infants took part in the Play Alone Conditions, two were in the Mimicry Condition and one infant was in the Non-Mimicry Condition) or during test (n = 2, one infant in the Mimicry-, the other in the Non-Mimicry Condition); fussiness (n = 2) or parental intervention (n = 1). Infants were assigned, as they became available, to one of the four experimental conditions until a final sample of 12 infants per group was reached. Infants were recruited from a list of local families who expressed interest in participating in studies of infant development. The study was approved by the Ethics Committee of Université Paris V. Families were middle- to upper-middle class. All parents provided written informed consent on behalf of the minors/children enrolled in the study before participating. Infants were randomly assigned to one of the four experimental groups:

- (1) Mimicry Condition (n = 12)
- (2) Non-Mimicry + Demo Condition (n = 12)
- (3) Play Alone + Demo Condition (n = 12)
- (4) Play Alone + No Demo Condition (n = 12)

### 2. Materials

Two sets of identical toys were used, including two large and two smaller plastic cups, two large plastic elephants, two doll figures, two coloured plastic balls, and two large plastic ducks. A different pool of 8 small attractive toys (average size of 3 cm×2 cm×2 cm) and a rake-like tool were used for demonstration and test on the Rake Task. The rake was a T-shaped object made of white cardboard; it was constructed for this experiment. The handle was 20 cm long and the head 20 cm wide. It was designed to be visually plain, so as not to distract infants. If during the sessions the infants did not show interest in a toy proposed for retrieval, it was replaced by another toy from the pool.

**Figure 1:**
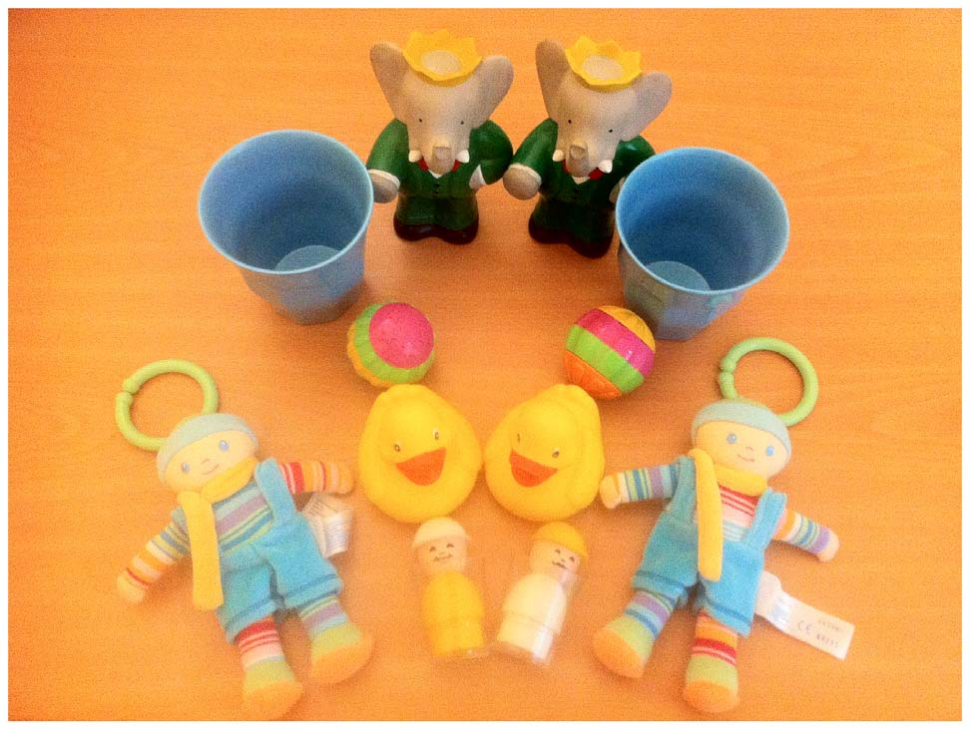
Materials. The double set of toys used for the Mimicry and Non Mimicry Conditions. One set only was used for the Play Alone Conditions where children played by themselves.

Fig. 1 illustrates these materials.

### 3. Procedure

Testing took place in the university laboratory. Before the session, the experimenters greeted the parents and informed them about the research. The sessions started once the infant was comfortable. We recorded the sessions on video for further analyses. Each session consisted of 3 phases, which were the following.

*Phase 1* (play) was different across our four conditions.

- (1) Mimicry + Demo Condition (n = 12): Infants were seated on the parent's lap in front of a table. Experimenter 1 (E1) sat across the table, opposite the infant. Experimenter 2 (E2) sat to the left of the infant and to the right of E1. The infant and E1 had identical sets of toys and E1 mimicked all the infant's actions on the toys, looking and smiling at the infant occasionally and commenting on the play (e.g.:“oh, this is nice”). During this time, E2 discussed and filled a general developmental information sheet with the parent regarding the infant. After 5 minutes of play, E2 put away all the toys and proceeded with *Phase 2*.
- (2) Non-Mimicry + Condition (n = 12): the setting and procedure was identical to the Mimicry + Demo Condition, with the only exception that E1 chose different toys as the infant and did not mimic the infant's actions (but she looked and smiled at the infant and commented on the play in the same way as in the Mimicry + Demo Condition).
- (3) Play Alone + Demo Condition (n = 12): the infant was seated at a small table and was allowed to play freely with one set of the same toys as in the above two conditions. During this time, the parent was seated with E1 and E2 at a separate table nearby to discuss and fill the same general developmental information sheet regarding the infant. After 5 minutes of play, E2 put away all the toys and proceeded with *Phase 2*.
- (4) Play Alone + No Demo Condition (n = 12): the setting and procedure was identical to the Play Alone + Demo Condition, with the difference that this time the experimenters proceeded immediately with *Phase 3.*

*Phase 2* (demonstration) was the same across conditions except for the baseline Play Alone + No Demo condition where the tool's use was not demonstrated. E1 and E2 remained in their places. E1 placed a toy on the table (from the new pool of 8 small attractive toys), out of E2's reach. E2 picked up the rake, placed near her, and used it to retrieve the toy with her right hand. She then grasped the toy with her left hand, looked at it with a happy expression and then put it down. This demonstration was repeated 5 times. Since E2, who produced rake action, was seated sideways, the infant could well observe from the side how the tool was used and how it contacted the target toy. At the end of the demonstration E1 left the room to avoid the effect of any affiliation with her on infants' performance.

*Phase 3* (test) was the same across the four conditions. E2, now seated across the table and was the only experimenter present. She placed the toy in front and out of reach of the infant, at a distance of approximately 70 cm from the infant. She then placed the rake near the infant's hand. Thus, from the infant's point of view, the toy was behind the rake and there was a large spatial gap between tool and toy. E2 then said:“Look at the (toy name); do you want to play with it? How can you get it?” The test ended after a 60s period starting when the infant first touched the rake or stretched his or her hand out toward the toy. If, within this test period, the infant became discouraged after having tried to retrieve the toy, failed, E2 encouraged the infant once by touching the toy and saying:“Go ahead; how can you get that (toy name)?”If the infant threw the rake away, E2 placed the rake near the infant once more and another 60s test period began. If the infant successfully retrieved the toy using the rake, the same toy was placed again in the same location for a new trial to ensure that the success was repeated. The actions infants produced during this test period were later scored for analyses. Parents were asked to restrain their infants if they tried to crawl onto the table to get the toy.

### 4. Data analysis

#### Infants' recognition of mimicry and play

In order to check whether infants' recognized that they were imitated in *Phase 1* of the Mimicry + Demo Condition, we coded the frequency of behaviors that indicate awareness of being imitated: (1) positive social signal (eye contact and smile or laughter), (2) alternates looks between experimenter's and own object, (3) tests experimenter's intention to imitate by changing action or object while looking at experimenter. If the infant produced two or more of these three behaviors, we considered that mimicry was recognized.

In order to check whether infants' recognized the adult's playful behaviour in *Phase 1* of the Non-Mimicry + Demo Condition, we coded the frequency of the following behaviours: (1) positive social signal (eye contact and smile or laughter), (2) gives toy to experimenter, (3) requests or takes toy from experimenter. If the infant produced two or more of these three behaviors, we considered that the experimenter was recognized as a social partner.

#### Infants' reproduction of the target action

Because full success is rare at this age [31], [32], each infant's behaviour was scored on a scale from 0 to 4 during the 60s test period of *Phase 3*. The scale was based on whether the infants manipulated one or both objects; did or did not make a connection between the toy and the rake without necessarily retrieving the toy; and whether they ultimately retrieved the toy using the rake.

*Score 0:* No try: grasps tool, discards it; looks at toy, looks at tool, and/or looks at the adult, doing nothing more.

*Score 1:* Interested in toy or tool alone: points to toy refusing or ignoring tool; grasps tool, discards it and points to toy; grasps tool and plays with it; grasps tool, swipes table with it, sweeping toy away by accident; grasps tool, plays with it and then rejects it, possibly interested in toy again.

*Score 2:* Interested in tool in connection with toy: grasps tool and touches or pushes toy with it.

*Score 3:* Interested in tool for retrieval, understands connection between the rake and the toy, but uses trial and error, therefore success is difficult or partial: grasps tool, makes clear attempts to bring toy closer, but fails or makes awkward movements to bring toy to hand and succeeds or retrieves toy after several attempts.

*Score 4:* Interested in tool for retrieval, solid understanding of connection between the rake and the toy, intentional full success: grasps tool directly, places it behind toy to retrieve it and succeeds.

An infant could perform one or several actions within the 60s manipulation period. Each action was scored. Infants were excluded (due to lack of interest in the experimental objects) if they only received score 0 during the 60s test period. In each condition, we analyzed the scores infants received for their first action (First score), for their highest scored action (Highest score) as well as the mean score of all their actions (Mean score).

Given the relatively small number of infants within each group, we also categorized their Highest scores into two categories for subsequent analysis. The first category included score 1 (toy and tool not contacted), and the second category included scores 2, 3 and 4 (toy and tool contacted).

#### Pointing

Pointing towards the toy during the 60s test period of *Phase 3* was coded in order to ascertain that the infant was interested in retrieving it. Each time the infant stretched his or her hand toward the toy either with an index finger or with the whole hand opened was coded as pointing.

#### Number of actions with rake

In order to assess the effect of mimicry on the frequency of rake actions, we calculated the number of all actions infants produced with the rake during the 60s test period of *Phase 3*.

#### Smiling, looking at experimenter's face and object while mimicked

In order to evaluate the emotional effect of mimicry, we coded the number of smiles infants produced during *Phase 1* while they played with the experimenter in the Mimicry and Non-Mimicry Conditions. As an indicator of infants' attention, we also recorded how many times they looked at the experimenter's face and her object in *Phase 1* while they played with the experimenter in the Mimicry and Non-Mimicry Conditions.

### 5. Scoring reliability

Infants' behaviours were coded from the videotapes, and 16 infants (33%) were coded independently by a second observer to assess inter-observer reliability. Both coders were blind to the experimental groups the infants belonged to. Reliability between the two observers was 90%.

## Results

### 1. Infants' recognition of mimicry and play

Ten infants (83%) in the Mimicry + Demo Condition produced a positive social signal during the mimicry phase and all infants (100%) produced the two other behaviours indicating recognition of being imitated: they alternated looks between experimenter's and own object and tested the experimenter's intention to imitate by changing action or object while looking at the experimenter.

All infants (100%) in the Non-Mimicry + Demo Condition produced all three behaviours (giving positive social signals, giving or taking toy), indicating recognition of the experimenter as a playful partner.

Thus, the responses of all infants could be retained for analysis.

#### First, Highest and Mean scores

**Figure 2:**
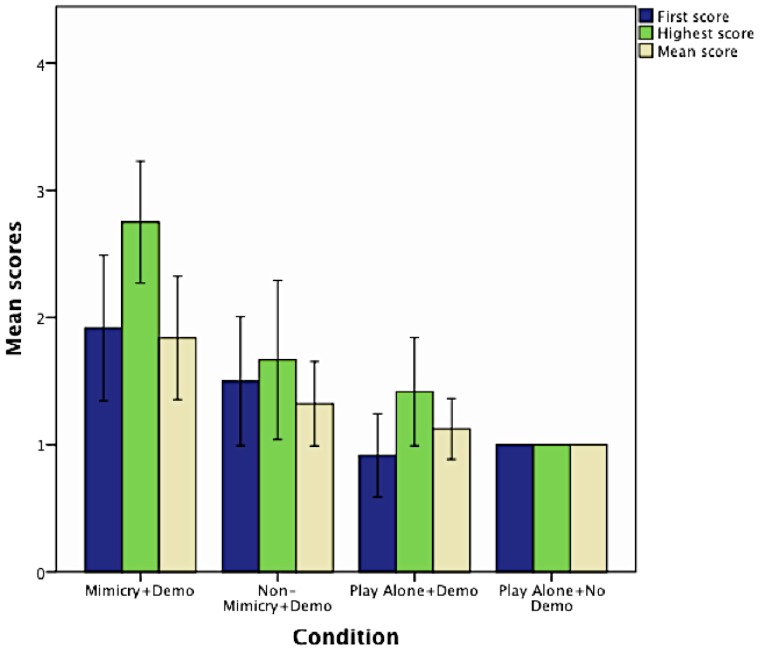
The effect of previous mimicry, play and demonstration on 16-month olds' tool use learning. Comparison of the means of infants' three score types obtained in the Rake Task across four conditions where they were either mimicked or not, played with an experimenter or played alone, saw a demonstration of the rake's use or not.

Fig. 2 represents the means of the three score types in each of the four conditions. A multivariate analysis of variance (MANOVA) was conducted to assess differences across conditions (independent variable: Condition) on the three score types (dependent variables: First score, Highest score and Mean score). A significant effect of Condition (F (9, 102)  = 4.37, p<.0001, partial η^2^ = .232) was found. Univariate tests showed that there were significant differences across conditions on First score, (F (3, 44)  = 6.1, p<.001, partial η^2^ = .294), Highest score (F (3, 44)  = 13.49, p<.0001, partial η^2^ = .479) and Mean score (F (3, 44)  = 6.56, p<.001, partial η^2^ = .310).

Multiple comparisons conducted with post-hoc LSD tests revealed the following effects at.05 level of significance.

#### The effect of mimicry

Table 1 compares infants' scores in the Mimicry + Demo Condition with scores obtained in the other three conditions (Non-Mimicry + Demo, Play Alone + Demo and Play Alone + No Demo). We can see that infants in the Mimicry + Demo Condition obtained significantly higher Highest scores and Mean scores than infants in any of the other three conditions. Their First scores were significantly higher than those of infants in the Play Alone + Demo and Play Alone + No Demo Conditions, but did not differ significantly from the First scores on infants in the Non-Mimicry + Demo Condition.

**Table 1:** The effect of previous mimicry on 16-month olds' tool use learning.

Dependent Variable	Condition		Sig.
First score	Mimicry+Demo	Non-Mimicry+Demo	,126
		Play Alone+Demo	**,001**
		Play Alone+No Demo	**,001**
Highest score	Mimicry+Demo	Non-Mimicry+Demo	**,000**
		Play Alone+Demo	**,000**
		Play Alone+No Demo	**,000**
Mean score	Mimicry+Demo	Non-Mimicry+Demo	**,015**
		Play Alone+Demo	**,001**
		Play Alone+No Demo	**,000**

Comparison of the means of infants' three score types obtained on the Rake Task in the Mimicry + Demo Condition with score means in the other three conditions, where infants were not mimicked, but played only with an experimenter (Non-Mimicry + Demo) or played alone before the demonstration (Play Alone + Demo) or again played alone, but did not see a demonstration (Play Alone + No Demo or Baseline). Bold numbers indicate significant differences between means (p<.05), as calculated with post hoc LSD tests.

Our first hypothesis was therefore confirmed; mimicry had a specific facilitating effect on infants' observational learning. Infants who were mimicked performed best on the Rake Task and obtained significantly higher scores than infants who played with the experimenter for the same amount of time or played by themselves. Interestingly, infants' First scores did not differ significantly in the Mimicry and Non-Mimicry conditions, showing that infants who were mimicked improved their performance during test.

#### The effect of play

Table 2 compares infants' scores in the Non-Mimicry + Demo Condition with scores obtained in the other three conditions (Mimicry + Demo, Play Alone + Demo and Play Alone + No Demo). Infants in the Non-Mimicry + Demo Condition received significantly higher First scores than infants in the Play Alone + Demo Condition. They also received significantly higher Highest scores than infants in the Play Alone + No Demo Condition. This shows that, although not as strongly as mimicry, playing with the infants without mimicking them also facilitated learning from demonstration, which, although for not all three score types, confirms our second hypothesis.

**Table 2:** The effect of previous play with a contingent partner on 16-month olds' tool use learning.

Dependent Variable	Condition		Sig.
First score	Non-Mimicry+Demo	Mimicry+Demo	,126
		Play Alone+Demo	**,034**
		Play Alone+No Demo	,068
Highest score	Non-Mimicry+Demo	Mimicry+Demo	**,000**
		Play Alone+Demo	,389
		Play Alone+No Demo	**,025**
Mean score	Non-Mimicry+Demo	Mimicry+Demo	**,015**
		Play Alone+Demo	,340
		Play Alone+No Demo	,122

Comparison of the means of infants' three score types obtained on the Rake Task in the Non-Mimicry + Demo Condition with score means in the other three conditions where infants were mimicked by the experimenter (Mimicry + Demo) or played alone before the demonstration (Play Alone + Demo) or again played alone, but did not see a demonstration (Play Alone + No Demo or Baseline). Bold numbers indicate significant differences between means (p<.05), as calculated with post hoc LSD tests.

#### The effect of demonstration

Table 3 compares infants' scores in the Play Alone + No Demo Condition with infants' scores in the other three conditions where infants saw a demonstration (Mimicry + Demo, Non-Mimicry + Demo Condition Play Alone + Demo and Play Alone + No Demo). Infants in the Play Alone + No Demo Condition scored significantly lower than infants in the Mimicry + Demo Condition on all three score types (First score, Highest score and Mean score). They also received significantly lower Highest scores than infants in the Non-Mimicry + Demo Condition. There were no significant differences between infants' scores in the Play Alone + No Demo and Play Alone + Demo Conditions. Thus, a demonstration was effective only when infants interacted with an experimenter beforehand, either just playing with her or with the experimenter mimicking them. When infants played by themselves in *Phase 1*, the demonstration had no effect, which confirms our third hypothesis.

**Table 3:** The effect of demonstration of tool's function on 16-month olds' tool use learning.

Dependent Variable	Condition		Sig.
First score	Play Alone+No Demo	Mimicry+Demo	**,001**
		Non-Mimicry+Demo	,068
		Play Alone+Demo	,757
Highest score	Play Alone+No Demo	Mimicry+Demo	**,000**
		Non-Mimicry+Demo	**,025**
		Play Alone+Demo	,154
Mean score	Play Alone+No Demo	Mimicry+Demo	**,000**
		Non-Mimicry+Demo	,122
		Play Alone+Demo	,543

Comparison of the means of infants' three score types obtained on the Rake Task in the Play Alone + No Demo Condition with score means in the other three conditions where infants saw a demonstration of the rake's function (Mimicry + Demo, Non-Mimicry + Demo and Play Alone + Demo). Bold numbers indicate significant differences between means (p<.05), as calculated with post hoc LSD tests.

#### Distribution of scores

**Figure 3:**
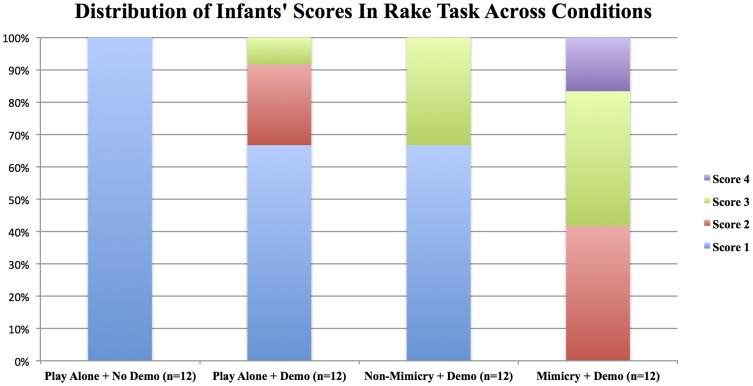
The effect of previous mimicry, play and demonstration on 16-month-old infants' best performance. Distribution of infants' Highest scores obtained in the Rake Task across four conditions where they were either mimicked or not, played with an experimenter or played alone, saw a demonstration of the rake's use or not.

Fig. 3 shows the distribution of infants' Highest scores across conditions. All infants (100%) in the Mimicry + Demo Condition made a connection between rake and toy (Highest score: 2, 3 and 4), whereas none of the infants (0%) did so in the Play Alone + No Demo Condition (scoring 1). In the Non-Mimicry + Demo and the Play Alone + Demo Conditions 8 of 12 infants (67%) connected rake and toy (Highest score: 2, 3 and 4). Fisher's exact tests show that infants were significantly more likely to connect rake and toy in the Mimicry + Demo Condition as compared with the Non-Mimicry + Demo and the Play Alone + Demo Conditions (both ps <.001).

### 3. Pointing

All except 2 infants pointed to the toy during test, which indicates that they were all motivated to retrieve it. The 2 infants who did not point or reach were both in the Mimicry + Demo Condition and both acted upon the toy with the rake (scoring either 2 or 3); therefore they also demonstrated interest towards the toy.

### 4. Number of actions with rake

We conducted nonparametric tests to assess the effect of mimicry on the frequency of actions infants produced with the rake. The Kruskal-Wallis test indicated a significant difference in the number of actions infants produced with the rake across the four experimental groups (χ^2^(3)  = 16.911, p = .001). Pairwise comparisons using the Mann-Whitney test revealed that this could be attributed to significant differences between the Mimicry + Demo and the Play Alone + No Demo Conditions (U(22)  = 8, p = .001) as well as the Play Alone + Demo and the Play Alone + No Demo Conditions (U(22)  = 16, p = .001). The mean number of actions infants produced in the rest of the conditions did not differ significantly.

This pattern of results suggests that witnessing a demonstration increased infants' propensity to produce actions with the rake. However, as there was no difference between the mean number of actions infants produced in the Mimicry + Demo- and the Non-Mimicry + Demo Conditions, we can conclude that being mimicked did not increase the frequency of rake actions infants produced.

### 5. Smiling, looking at experimenter's face and object while mimicked

**Figure 4:**
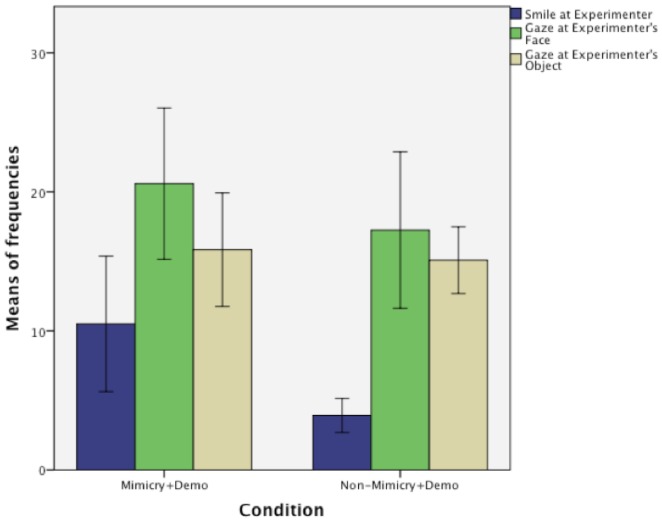
The effect of mimicry on 16-month-old infants' smiling and looking behaviour. Comparison of the frequencies at which infants smiled at the Experimenter and looked at her face or at the object she was holding in the Mimicry and the Non-Mimicry + Demo Conditions.

Fig. 4 shows the mean frequencies at which infants smiled at the experimenter, looked at her face or at the object she was holding in the Mimicry and the Non-Mimicry + Demo Conditions. Infants smiled significantly more at the experimenter in the Mimicry + Demo Condition (M_Mimicry/Smile_  = 10.5, M_Non-Mimicry/Smile_  = 3.9, t(22)  = 2.88, p<.01), indicating that mimicking the infants triggered more positive emotions than playing with them without mimicry. Infants looked at the experimenter's face and her object equally in the Mimicry and the Non-Mimicry + Demo Conditions (M_Mimicry/Face_  = 20.58, M_Non-Mimicry/Face_  = 17.25, t(22)  = 0.94, p = .36, ns and M_Mimicry/Object_  = 15.83, M_Non-Mimicry/Object_  = 15.08, t(22)  = 0.35, p = .73, ns). This indicates that there were no differences between these two groups in the amount of attention directed towards the experimenter or her object.

## Discussion

Despite the essential role of spontaneous mimicry in infants' daily lives [1], [16], [17] and mimicry's social effects [1], [9], [10], [13], [14], [16], [17], the effect of mimicry on social learning has not yet been directly investigated. Therefore, the aim of this study was to explore whether mimicry affects how infants learn by observation a novel tool use action.

Our first hypothesis was confirmed, as infants who were mimicked beforehand learned best from a demonstration of the rake's use and performed better on a task requiring the use of the rake than infants who played with the experimenter without mimicry or played by themselves before the demonstration.

Our second hypothesis was partly confirmed. When compared with infants who played by themselves, infants who played with a socially contingent experimenter without being mimicked learned better from a demonstration. However, the difference in scores reached significance only for the score of the first action.

Finally, in line with previous results [24], [25], infants did not learn from demonstration of the rake's use when they had no previous interaction with the experimenter (either mimicry or play), which confirmed our third hypothesis.

Interestingly, the number of actions infants produced with the rake following the demonstration was similar in the mimicked group and the group who played alone beforehand. The demonstration itself therefore triggered a general interest in the rake through stimulus enhancement. Producing the same number of actions with the rake however, did not necessarily bring about success, as infants who were also mimicked beforehand produced actions that scored significantly higher. In other words actions were of comparable quantity for these two groups, their quality however was different.

Thus, we show that mimicry influences how infants subsequently process and learn from a situation. Similar cognitive effects of mimicry have been observed in adults. Van Baaren et al. [26] explored the relation between behavioral mimicry and field-dependent versus field-independent processing style [27]. They found that participants who were mimicked subsequently processed information in a more field- or context-dependent manner (succeeding more in identifying an embedded figure) as compared to participants who were not mimicked. Furthermore, mimicry has been associated with a persuasive effect and compliance toward the mimicker's suggestions [28], even when the mimicker was a digital avatar [29]. What may be the mechanisms driving this powerful learning effect of mimicry?

One might propose that infants who were mimicked simply continued the imitative game and reproduced the experimenter's tool use action blindly, without having learned about the rake's function. If this were the case, then infants would not have produced alternative strategies, such as pointing towards the toy and trying to retrieve it with the hand. The pattern of infants' actions suggests instead that infants who were mimicked improved their performance during test and used the rake once their own strategy failed. This demonstrates their understanding of the fact that rake's function is to solve the problem of retrieving an out-of-reach object. Studies on infants' selective or rational imitation also support this view [30], [31].

The two mentioned studies that systematically investigated the social effects of mimicry proposed that affiliative orientation [9] or social bonding [10] might be the mediating factor. In our study however, social bonding could not have driven the effects, as different experimenters mimicked and tested the infants. The possibility remains though that the affiliative orientation induced by mimicry had a general effect that resulted in learning from others (not the mimicking person), in the same way as infants also helped adults that did not mimic them earlier [9].

A second possibility is that the positive mood induced by mimicry drove the learning effect. Indeed, infants who were mimicked smiled significantly more and displayed positive emotions more frequently than infants who only played with the experimenter in our study. We have seen that there were no differences between the two groups in the number of times infants looked at the experimenter's face or the object she was holding. Although we could not grasp any differences in attention by comparing looking frequencies, it remains possible that infants who smiled when mimicked were also more attentive during the subsequent demonstration, due to a higher level of arousal or motivation. In the two earlier studies no differences in mood were observed, however, mood was not measured directly, as the experimenter's general impression [10] or parental report [9] was considered. Indeed, it has been shown that a positive emotional state shifts children toward a more global mode of perception. Poirel et al. [32] placed 5-year-olds (known to have a local perceptual bias) and 8-year-old children (known to pay attention predominantly to global information) in either a neutral or pleasant emotional context and subsequently presented them with a global/local visual judgment task. Following exposure to emotionally pleasant pictures, there was a global shift, at both ages, to a perceptual bias toward global information. The authors concluded that emotion might strongly affect children's visual perception. In our tool use task, in order to succeed at retrieving the toy, infants needed to consider the two objects together (tool and toy). Thus, infants' better performance following mimicry may have been driven by such a perceptual bias toward global information, whereas infants in other conditions remained in their preferred local perceptual processing mode (considering tool and toy separately), which, in this case, did not help to solve the retrieval problem. In order to confirm this possibility, it would be interesting to use a global/local judgment task in young infants to see whether, like 5-year-olds, 16-month-olds are also biased toward local perception. This could explain their difficulties in relating tool and toy to each other. Note that the perceptual bias toward global information described in Poirel et al.'s [32] study is similar to the bias toward the field dependent cognitive processing style (where objects are perceived within their context rather than separately) observed in adults following mimicry [26]. Therefore in adults as well, this effect of mimicry may well be driven by the positive emotional state induced by mimicry. Indeed, positive emotions have been shown to improve creative problem solving and facilitate cognitive flexibility in adults [33], which in turn has been associated with an increase in brain dopamine levels resulting from positive emotions [34].

How does mimicry induce positive emotions? Mimicry involves an exaggerated form of social contingency, which may have been particularly appreciated by infants [8]. In Carpenter et al.'s [22] study temporal contingency was systematically controlled for, but temporally contingent behavior without mimicry (the experimenter performed a different action each time) did not have an effect on infants' prosocial behavior. Temporal contingency did not explain the preference of a mimicking adult either in 14-month-olds [13]. Social contingency and mimicry however, involves both temporal and spatial contingencies, the latter corresponding to a matching of object-related actions in the spatial domain. It is therefore possible that the presence of both contingencies induced the positive emotions that in turn mediated the facilitating effect of mimicry on learning (and to a lesser extent, the facilitating effect of socially contingent play).
